# Rapid coral mortality following unusually calm and hot conditions on Iriomote, Japan

**DOI:** 10.12688/f1000research.12660.2

**Published:** 2018-03-12

**Authors:** Andrew H Baird, Sally A. Keith, Erika Woolsey, Ryuta Yoshida, Tohru Naruse

**Affiliations:** 1 ARC Centre of Excellence for Coral Reefs Studies, James Cook University, Queensland, Australia; 2 Lancaster Environment Centre, Lancaster University, Lancaster, UK; 3 Centre for Macroecology, Evolution and Climate, Natural History Museum of Denmark, University of Copenhagen, Copenhagen, Denmark; 4 The Hydrous, San Francisco , CA, USA; 5 Tateyama Marine Laboratory, Marine and Coastal Research Center, Ochanomizu University, Tateyama, Chiba, 294-0301, Japan; 6 Iriomote Station, Tropical Biosphere Research Center, University of the Ryukyus, Taketomi, Okinawa, Japan

**Keywords:** climate change, coral bleaching, coral reefs, disturbance

## Abstract

Coral bleaching can be induced by many different stressors, however, the most common cause of mass bleaching in the field is higher than average sea surface temperatures (SST). Here, we describe an unusual bleaching event that followed very calm sea conditions combined with higher than average SST. Patterns of mortality differed from typical bleaching in four ways: 1) mortality was very rapid; 2) a different suite of species were most affected; 3) tissue mortality in *Acropora* spp. was often restricted to the center of the colony; 4) the event occurred early in summer. The two weeks prior to the event included 8 days where the average wind speed was less than 3 ms ^-1^. In addition, SSTs in the weeks preceding and during the event were 1.0-1.5°C higher than the mean for the last 30 years. We hypothesize that this unusual bleaching event was caused by anoxia resulting from a lack of water movement induced by low wind speeds combined with high SST.

## Introduction

Coral bleaching is a generalized response that can be induced by many different stressors ^1– 3^. Whilst the most common cause of large scale bleaching on coral reefs is unusually high sea surface temperatures (SSTs) ^4, 5^, prolonged periods of calm weather have also been associated with mass bleaching events in the Caribbean ^6, 7^ and the Indo-Pacific ^8– 10^. Experimental work has also confirmed that low water flow can exacerbate thermal bleaching ^11, 12^.

The ecology of thermal coral bleaching in response to high SSTs is reasonably well documented. For example, colonies affected by high temperatures typically take between two to six weeks to bleach and bleached tissue can take another two to twenty weeks to die ^13^. In addition, species vary in their susceptibility to thermal bleaching ^14, 15^, resulting in a predicable hierarchy of response ^16, 17^. Temporal patterns are also apparent with most high temperature induced mass bleaching events generally occurring towards the end of the summer months ^18, 19^. Any change in this predictable bleaching ecology suggests an alternative cause (i.e., not thermal stress) for a given bleaching event.

Here, we describe an atypical bleaching event that we hypothesize was caused by an interaction of temperature with very calm sea conditions caused by an extended period of low winds. We identify a number of characteristic features of this calm weather bleaching that allow it to be distinguished from thermal bleaching in the field. Establishing the cause of specific bleaching events is vital in order to correctly attribute damage caused by climate change and other potential stressors.

## Methods

The study site was on the reef crest (1 m depth) at Nata Reef, Iriomote, Japan (24.4282°N, 123.7955°E). Initial observations at the site were made between 26 and 29 May, 2016 at which point in time no bleached corals were noted. Surveys to quantify bleaching and mortality were conducted on 12 June, 2016. Twenty replicate 1m ^2^ quadrats were placed haphazardly on the reef crest, and the condition and species identity of all hard coral colonies with a maximum diameter greater than 5cm were recorded. Species were identified in the field following ^20^ and the names updated to the currently accepted names following ^21^ Colonies were placed in one of six bleaching categories following ^22^: (1) unbleached, (2) the entire colony pale, (3) 1–50% of the colony white, (4) 51–99% of the colony white, (5) 100% of colony white or fluorescent, or (6) recently dead. The data from the quadrats was pooled as the data was collected. The bleaching mortality index was calculated following ^16^. Data on environmental conditions leading up to the bleaching episode and for a similar time frame in 2015 were obtained from the Japan Meteorological Agency, which allows for these data to be used as long as due credit is given.

## Results

Bleaching and mortality was rapid. No colonies were bleached at the time of the first surveys (26 May, 2016) yet two weeks later (12 June, 2016), 5% of colonies were dead and a further 31% were bleached ( Table 1).

**Table 1.** Bleaching categories of hard corals at Nata Reef on 12 June 2016. BMI = Bleaching Mortality Index.

taxa	unbleached	moderate	severe	dead	BMI	n
*Acropora selago*	0	0	0	100	100	1
*Montipora aequituberculata*	0	0	0	100	100	3
*Montipora efflorescens*	0	27	27	45	73	11
*Goniastrea pectinata*	0	50	50	0	50	2
*Milleporidae*	17	33	50	0	44	6
*Dipsastraea rotumana*	0	100	0	0	33	1
*Montipora turgescens*	0	100	0	0	33	1
*Platygyra ryukyuensis*	25	50	25	0	33	4
*Platygyra verweyi*	67	0	0	33	33	3
*Dipsastrea pallida*	30	50	20	0	30	10
*Montipora crassituberculata*	46	32	18	4	26	28
*Montipora digitata*	71	0	29	0	19	7
*Acropora nasuta*	50	50	0	0	17	2
*Pocillopora damicornis*	67	22	11	0	15	9
*Pavona venosa*	57	43	0	0	14	7
*Porites annae*	60	40	0	0	13	5
*Acropora hyacinthus*	71	29	0	0	10	7
*Platygyra pini*	75	25	0	0	8	4
*Porites cylindrica*	77	23	0	0	8	13
*Acropora digitifera*	81	19	0	0	6	32
*Galaxea fascicularis*	82	18	0	0	6	11
*Favites halicora*	86	14	0	0	5	7
*Goniastrea retiformis*	86	14	0	0	5	14
*Acropora aspera*	100	0	0	0	0	1
*Acropora gemmifera*	100	0	0	0	0	1
*Astrea annuligera*	100	0	0	0	0	1
*Cyphastrea serailia*	100	0	0	0	0	3
*Favites abdita*	100	0	0	0	0	3
*Favites magnistellata*	100	0	0	0	0	2
*Montipora monasteriata*	100	0	0	0	0	4
*Pavona decussata*	100	0	0	0	0	2
*Porites lichen*	100	0	0	0	0	3
*Porites lutea*	100	0	0	0	0	1
*Porites rus*	100	0	0	0	0	6
*Psammocora contigua*	100	0	0	0	0	1
total	64	23	8	5	216	18

**Figure 1.**
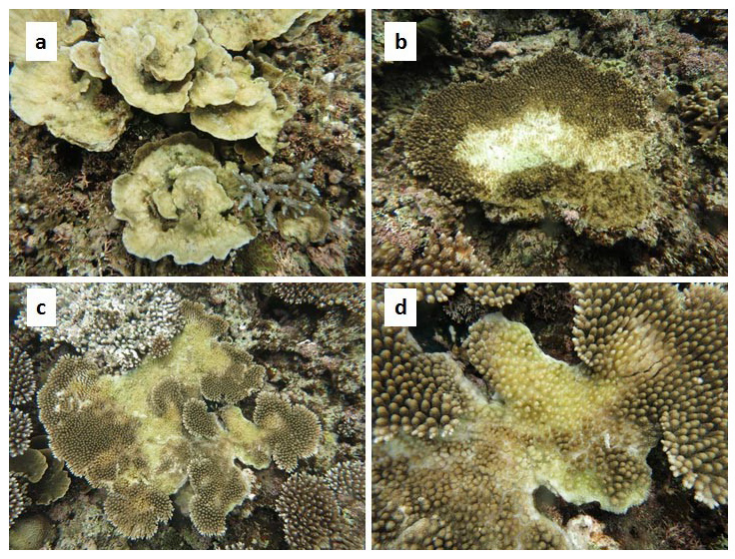
( **a**) Dead and dying *Montipora aequituberculata* colonies ( **b**) *Acropora hyacinthus* colony with bleached and dying tissue in the middle of the colony ( **c**) a second *A. hyacinthus* colony ( **d**) close up of the colony in ( **c**). Images were captured using a Canon S100 digital camera in waterproof housing.

Mortality was highest in *Montipora aequituberculata* and *M. efflorescens* ( Figure 1A), ** and in an additional three species of the family Merulinidae, that were also badly affected ( Table 1). Bleaching and tissue mortality were generally restricted to the center of colonies in the locally abundant species *Acropora digitifera* and *A. hyacinthus* ( Figure 1B, C, D).

The bleaching event occurred early in June, the first month of the northern summer, following a period of low wind and higher than average sea surface temperature (SST). Eight days in the previous two weeks had average wind speeds of under 3 ms ^-1^ ( Table 2). Winds were also mostly from the south, which is offshore at the study site and therefore likely to further reduce wave size and water motion ( Table 2). Mean daily SSTs in the month preceding the second survey were 0.0–1.5°C higher than the mean for the previous 30 years ( Table 3). Wind speeds were higher and SST lower during the same time interval in 2015 ( Table 2 & Table 3).

**Table 2.** Mean daily wind speeds in the 12 days prior to the first observations of bleaching on 12 June 2016 and for the same dates in 2015. Data from Japan Meteorological Agency.

Date	2015 wind speed (m/s)	2015 wind direction	2016 wind speed (m/s)	2016 wind direction
30 May	2.2	SSE	2.1	ENE
31 May	3.1	ENE	1.8	SE
1 June	2.6	NE	3.4	W
2 June	4.8	SSW	3	NE
3 June	4.6	SSW	2.1	SW
4 June	2.3	S	2.1	ENE
5 June	5.5	SSW	3.2	NE
6 June	3.8	S	2.5	NE
7 June	4.2	SSW	1.6	SE
8 June	3.3	SW	1.9	ESE
9 June	3	SSW	2	ENE
10 June	4.2	SSW	3	SSW
11 June	5.3	S	6.4	SSW
12 June	4	S	8.2	SSW

**Table 3.** Sea surface temperature anomalies in the weeks preceding the bleaching event on Nata Reef and a similar time interval in 2015. Values are the degrees in centigrade above the 30 year average for this site in each time interval. Data from the Japan Meteorological Agency.

10 day period ending	2015 SST anomaly °C	2016 SST anomaly °C
10 April	0	0
20 April	-0.5	1
30 April	0	1.5
10 May	0.5	1.5
20 May	0.5	1
30 May	0.5	1
10 June	0.5	1

## Discussion

This bleaching event was different to typical thermal bleaching in a number of important ways. In particular, rapid tissue mortality, an atypical hierarchy of susceptibility, and the occurrence of the event in early summer, all distinguish this event from typical thermal bleaching. We hypothesize that unusually high SST combined with a lack of water flow due to low winds speeds resulted in anoxic stress to these colonies. This hypothesis is supported by very low wind speeds ( Table 2) combined with higher than average mean daily SST ( Table 3) in the weeks prior to the event.

In contrast to the typical thermal response, bleaching and mortality were very rapid, with a high proportion of colonies bleached and some dying within the two week period between the surveys ( Table 1). Bleaching and, in particular, mortality typically take between 4–6 weeks to present in corals following thermal stress ^13^. In addition, the hierarchy of susceptibility was very different to that following thermal bleaching. Here, the worst affected species included two *Montipora* spp. and a number of merulinids ( Table 1), when typically *Acropora* spp. and *Pocillopora* spp. are the most severely affected following thermal bleaching ^5, 15, 22^.

The pattern of tissue bleaching and mortality was also unusual. In *Acropora* colonies the typical pattern following thermal stress is for the whole colony to bleach ^13^. In contrast, mortality was restricted to the center of most *Acropora* colonies in this event ( Figure 1a, b, c). Tissue mortality beginning in the center of the colony is suggestive of anoxia, which often occurs in aquaria with inadequate flow or oxygenation (pers obs). This pattern of mortality is also superficially similar to feeding scars caused by *Acanthaster planci* or *Drupella* spp. ^23^ and a naïve observer might well have attributed this mortality to either of these corallivores ^24^. A thorough search of the site, including underneath these and adjacent colonies, indicated that neither of these corallivores were present.

The timing of the bleaching event in early summer is also unusual. Thermal bleaching typically occurs much later in the summer. For example, recurrent seasonal bleaching on Magnetic Island, Australia, occurs in the last month of the austral summer i.e., February ^18^. Similarly, the 1998 mass bleaching event in Japan was first noticed in the latter part of the summer i.e., late July ^25^. In contrast, this calm weather event occurred early in June, the first month of the northern summer.

Doldrums-like conditions (defined by NOAA as days with average wind speeds of less than 3 ms ^-1^) have previously been linked to mass bleaching events ^6– 9^. However, the capacity of calm weather to cause more localized damage outside of the typical thermal bleaching window in late summer has not previously been recognized. In addition, the potential link to anoxia, while tested in the laboratory ^26^, has not been made in the field. This observation is especially important in the context of the continuing increase in the scale and frequency of mass bleaching events ^27^ because it would generally be assumed that this small-scale phenomenon might presage a larger mass bleaching event. Determining the cause of specific bleaching events is vital in order to accurately distinguish the effects of climate change versus other causes of degradation on coral reefs.

## Data availability

The pooled raw bleaching data is provided in Table 1.

Source data for Table 2 are available from the Japan Meteorological Agency, at:

http://www.data.jma.go.jp/obd/stats/etrn/view/daily_s1.php?prec_no=91&block_no=47917&year=2015&month=05&day=30&view=a3

http://www.data.jma.go.jp/obd/stats/etrn/view/daily_s1.php?prec_no=91&block_no=47917&year=2015&month=06&day=30&view=a3

http://www.data.jma.go.jp/obd/stats/etrn/view/daily_s1.php?prec_no=91&block_no=47917&year=2016&month=05&day=30&view=a3

http://www.data.jma.go.jp/obd/stats/etrn/view/daily_s1.php?prec_no=91&block_no=47917&year=2016&month=06&day=30&view=a3

Source data to generate the values in Table 3 are available from the Japan Meteorological Agency, at: http://bit.ly/2y8qlBw.
