# Supplementary material for: Rapid coral mortality following unusually calm and hot conditions on Iriomote, Japan
Source: F1000Res. 2018 Mar 12;6:1728. Originally published 2017 Sep 22. [Version 2] doi: 10.12688/f1000research.12660.2 (PMC5887074; doi:10.12688/f1000research.12660.2)
Supplement: Peer review history

# Peer review: Referee response for version 2

**DOI:** 10.5256/f1000research.15488.r31834

**Published:** 2018-04-05

**Authors:** Mikhail V. Matz

**Affiliations:** 1 Department of Integrative Biology, University of Texas at Austin, Austin, TX, USA

I’m generally satisfied with the authors' response, although I really hoped to see more previous-years data than just one year before the event - in my opinion the case for the “doldrums effect” becomes progressively more convincing the better you can show that these conditions were indeed really unusual. Also, next time, please consider using some form of graphs rather than tables to illustrate this type of data (perhaps a scatterplot of wind speed vs temperature, points colored by month?... Or, overlaid temperature and wind speed line graphs for the event time vs previous years?)

I have read this submission. I believe that I have an appropriate level of expertise to confirm that it is of an acceptable scientific standard.

# Peer review: Referee response for version 1

**DOI:** 10.5256/f1000research.13707.r26305

**Published:** 2017-12-04

**Authors:** Robert van Woesik

**Affiliations:** 1 Department of Biological Sciences, Florida Institute of Technology, Melbourne, FL, USA

The manuscript by Baird and colleagues is a useful contribution to the literature. There are however a few minor edits that are necessary to make this short contribution publishable.

 Firstly, the title is inaccurate, or at least misleading. Doldrums is a maritime term that refers to the low-pressure area affected by the Intertropical Convergence Zone, where the prevailing winds are generally calm. The doldrums are considered to lie between 10 ^o^N and 10 ^o^S. Using the term doldrum-like conditions, instead of calm conditions, for a reef that is located at latitude 24 north confuses the terminology. I understand that NOAA, the US agency, has been using the term to refer to atypically calm periods irrespective of latitude, but again that doesn’t make it correct because they are also misusing the term. Change the title to reflect a short, atypically calm period.

 Table 1 should not simply have the corals in alphabetical order, but instead sort the table from most to least impacted.

 Introduction

 Again, revise the use of the term doldrums.

 Methods

 Several periods are missing at the ends of sentences in the Methods section.

 Specify the aspect of the site. Which direction is the site facing?

 Data were pooled and were collected, not data was.

 Results

 Delete the word “who” in the sentence “who were also badly affected (Table 1)”

 Discussion

 “was different from…”, not different to

 Revise doldrums-like conditions in several places.

 Again, different from, not different to.

 Periods are missing from the ends of sentences in several places of the Discussion.

 Split infinitive: change to : has not been previously…

 Data availability

 Data are provided, not, data is provided

I have read this submission. I believe that I have an appropriate level of expertise to confirm that it is of an acceptable scientific standard.

# Peer review: Referee response for version 1

**DOI:** 10.5256/f1000research.13707.r26307

**Published:** 2017-10-05

**Authors:** Mikhail V. Matz

**Affiliations:** 1 Department of Integrative Biology, University of Texas at Austin, Austin, TX, USA

This is a compact report of an unusual bleaching episode, presenting an interesting hypothesis that it could have been caused by anoxia during atypical doldrums conditions. Both are valuable for improving our understanding of factors affecting coral survival in times of changing climate.

 While there is no way to rigorously prove that the doldrums hypothesis is true, it certainly seems reasonable (and the wording throughout the paper is appropriate – there is never a claim that doldrums actually caused the observed bleaching).

 That said, the paper would greatly benefit from expanded characterization of the doldrums episode, beyond tables 1 and 2. Is it possible to make wind speed and temperature graphs for broader range of dates (start in April) and compare that to conditions in the same time in previous years? This might show what was actually the most unusual – early temperature anomaly, low wind anomaly (by the way absolute values in Table 2 are not very informative without knowing how much below typical they are), or a combination of the two? Come to think of it, wave height could be a better proxy of water movement on the reef than wind speed, if it is possible to get that data (from satellite?).

 Minor things:

 “Tissue mortality beginning in the center of the colony typically indicates anoxia, which often occurs in aquaria with inade- quate ow or oxygenation (pers obs)” – I am not quite happy with the word “typically” used in a statement supported by nothing but personal observation… Can you please try once more to look up literature on this? (this is why my answer in "partly" to the literature citing question). This would also strengthen the doldrums case.

 “The bleaching mortality index was calculated following [16]” – as far as I can see, this index is not actually used for anything in the paper, so maybe it is not necessary? If keeping it, please expand a little: “The bleaching mortality index was calculated by [doing this and that], following [16].”

I have read this submission. I believe that I have an appropriate level of expertise to confirm that it is of an acceptable scientific standard, however I have significant reservations, as outlined above.
